# Concentration-Dependent Efficacy of Recombinant Human Bone Morphogenetic Protein-2 Using a HA/β-TCP Hydrogel Carrier in a Mini-Pig Vertebral Oblique Lateral Interbody Fusion Model

**DOI:** 10.3390/ijms24010892

**Published:** 2023-01-03

**Authors:** Hye-Yeong Lee, Ji-In Kang, Hye-Lan Lee, Gwang-Yong Hwang, Keung-Nyun Kim, Yoon Ha

**Affiliations:** 1 Spine & Spinal Cord Institute, Department of Neurosurgery, College of Medicine, Yonsei University, Seoul 03722, Republic of Korea; 2 Department of Neurosurgery, Yongin Severance Hospital, Yonsei University College of Medicine, 363, Yongin-si 16995, Republic of Korea; 3 POSTECH Biotech Center, Pohang University of Science and Technology (POSTECH), Pohang 37673, Republic of Korea

**Keywords:** recombinant human bone morphogenetic protein-2, HA/β-TCP hydrogel putty, oblique lateral interbody fusion model

## Abstract

Bone morphogenetic protein-2 (BMP-2) is used in the treatment of degenerative spinal disease and vertebral fractures, spine fusion, dental surgery, and facial surgery. However, high doses are associated with side effects such as inflammation and osteophytes. In this study, we performed spinal fusion surgery on mini-pigs using BMP-2 and a HA/β-TCP hydrogel carrier, and evaluated the degree of fusion and osteophyte growth according to time and dosage. Increasing the dose of BMP-2 led to a significantly higher fusion rate than was observed in the control group, and there was no significant difference between the 8-week and 16-week samples. We also found that the HA + β-TCP hydrogel combination helped maintain the rate of BMP-2 release. In conclusion, the BMP-2-loaded HA/β-TCP hydrogel carrier used in this study overcame the drawback of potentially causing side effects when used at high concentrations by enabling the sustained release of BMP-2. This method is also highly efficient, since it provides mineral matter to accelerate the fusion rate of the spine and improve bone quality.

## 1. Introduction

Bone morphogenetic proteins (BMPs) are multifunctional growth factors belonging to the transforming growth factor–β superfamily that were discovered during the demineralization process of cortical bone, and are potent factors that induce bone and cartilage formation and bone regeneration [1,2,3,4,5]. BMP is currently used in various applications, such as the treatment of degenerative spinal disease and vertebral fractures, spine fusion, dental surgery, and facial surgery. BMP-2 and BMP-7 are osteoinductive adjuvants widely used in surgery. Both have been approved by the United States Food and Drug Administration. BMP-7 has been utilized for long bone defects, revisional posterolateral fusion, and long bone malunion surgery. BMP-2 has also been approved for anterior lumbar interbody fusion, open tibial fractures (2004) and oral maxillofacial reconstruction (2007) [6]. Spine surgeons have used BMP-2 for anterior cervical fusion as an off-label application, and the surgery showed a good fusion rate and a good prognosis. However, serious side effects, such as airway narrowing and death due to soft tissue inflammation and swelling, have also been reported [4,5,7,8].

BMP-2 is secreted and binds to its receptor, BMP-2 receptor type I/II, either activating the canonical pathway via Smad1/5/8 or promoting mitogen-activated protein kinase (MAPK) pathway activation [9,10,11]. Among the BMP target genes, these signaling pathways increase the expression of the RUNX2, Dlx5, and osterix transcription factors, which are related to osteogenesis; this, in turn, increases the expression of the osteocyte differentiation factors ALP, IGF-1, osteopontin, and osteocalcin, and resultingly promotes osteogenesis [9,10,11]. However, BMP-2 was also found to a promote bone resorption processes that antagonize bone formation. The expression of the SOST and DKK1 genes is increased through the osteogenic mechanism Smad, and these two factors bind to the Wnt pathway receptor LRP5 and inactivate the Wnt pathway. This action increases the RANKL/OPG ratio and promotes osteoclast activation, ultimately resulting in bone mass loss [12]. The ambivalence of the bone metabolism regulation mechanism of BMP-2 suggests that an elaborate regulatory system exists in vivo to prevent excessive bone formation. Indeed, clinical trials using supraphysiological doses of recombinant human BMP-2 (rhBMP-2) in milligrams for anterior lumbar interbody fusion have demonstrated bone fusion rates superior to those achieved with standard iliac bone grafts [3,13,14]. Therefore, using small amounts of BMP-2 may be sufficiently effective, as the use of high doses of rhBMP-2 cause side effects including inflammation, soft tissue edema, intestinal edema, and unintended ectopic bone formation, which could impair its extensive clinical application [15,16]. 

Since BMPs are readily diffused and cleared from the body, it has been concluded that using mediators that can be slowly and sustainably released so that they can act on target cells in a prolonged manner is more beneficial over immediate release for bone formation [17]. A transporter (carrier) must bind BMPs well and have good biocompatibility. Ideally, a transporter could be reabsorbed, lost, or integrated with bone during osteogenesis. Many delivery vehicles have been proposed, including gelatin, collagen sponges, poly-L-lactic acid scaffolds, hydroxyapatite (HA), hyaluronic acid, and fibrin gels. However, the BMP-release morphology of these delivery systems was not ideal, exhibiting a high initial release burst over a short period of time [18,19,20,21]. Therefore, we have investigated the use of a carrier that provides sustained release of BMP-2 to overcome this drawback. In addition, this carrier is easy to handle and has other desirable properties [15,16,22,23], including good mechanical strength and spatial control of new bone formation [24,25,26,27]. The carrier under discussion is a system based on a ceramic/polymer composite material that has received particular attention as a sustained-release BMP-delivery system. That is, HA provides mechanical strength and a β-tricalcium phosphate (TCP) hydrogel (TH) provides excellent handling while ensuring a sustained release of rhBMP-2 [15,28]. Furthermore, we expected additional effects on osteogenesis through the use of two types of ceramics with different levels of biodegradability [29,30]. Poorly biodegradable HA granules prevent soft tissue invasion and allow the scaffold volume to be maintained during osteogenesis, while the TH can be gradually degraded, reabsorbed, and efficiently replaced by new bone [29,31]. In addition, the mix of the two different ceramics allow the surrounding cells to sequentially secrete the rhBMP-2 that had been taken up during degradation after transplantation into the fusion site, and also provide sufficient space for full osteoblast transportation, thereby conferring greater strength for the newly formed bone tissue [8]. 

In this study, we investigated the in vitro release kinetics of rhBMP-2 from the developed ceramic/polymer composites and the in vivo concentration-dependent fusion effect of rhBMP-2. Furthermore, in order to comparatively analyze the effective range of application of rhBMP-2, we constructed a mini-pig spinal fusion model that has the same structural morphology as the human spine and shows a clinical response using the same method as in clinical practice, observed at 8 weeks and 16 weeks.

## 2. Results

### 2.1. Advantages of Ceramic Carriers and Hydrogel Polymer Mixture for Sustained Release of Growth Factors

**Figure 1:**
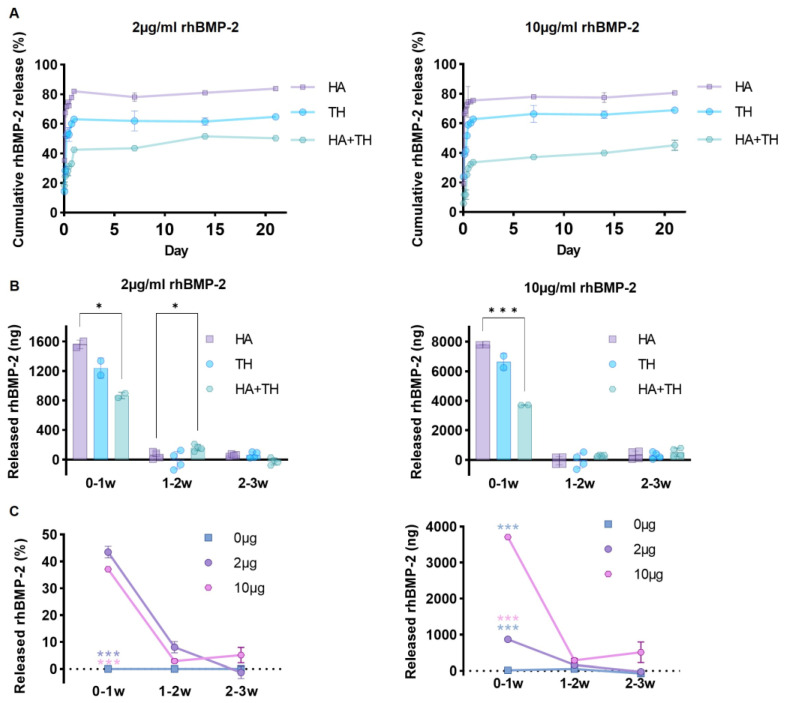
In vitro release kinetics according to the composition of the carrier and the concentration of rhBMP-2. (**A**) The release pattern of 2 μg and 10 μg rhBMP-2 from HA, TH, and HA + TH using ELISA. Mixing rhBMP-2 at a high concentration with HA + TH as a carrier delayed the release of rhBMP-2. (**B**) The amount of rhBMP-2 released at intervals of 0–1, 1–2, and 2–3 weeks. From 0 to 1 week, HA and TH released much more rhBMP-2 than HA + TH. (**C**) Concentration-dependent release patterns of rhBMP-2 from HA + TH. Although there was a difference in the amount of rhBMP-2 released depending on the rhBMP-2 concentration, the pattern was almost the same. Most of the rhBMP-2 was released in 0–1 weeks, and there was no significant change at 1–2 weeks. However, when 10 μg of rhBMP-2 was used, it was detected at 2–3 weeks in amounts greater than observed at 1–2 weeks, along with carrier degradation. * *p* < 0.05 and *** *p* < 0.001 indicate statistically significant differences.

To confirm the in vitro release kinetics depending on the carrier composition and concentration of rhBMP-2, the amount of rhBMP-2 released from HA, TH, and HA + TH was evaluated using ELISA (Figure 1A). Specifically, 2 μg of rhBMP-2 was mixed with a carrier and observed for up to 3 weeks. The amount of rhBMP-2 released was 8061.33 ng (80.61%) in the HA group, 6888.07 ng (68.88%) in the TH group, and 4512.65 ng (45.13%) in the HA + TH group. When 10 μg of rhBMP-2 was mixed, 1676.27 ng was released in the HA group, 1295.08 ng in the TH group, and 1004.18 ng in the HA + TH group. These results indicated that using HA + TH was ideal for the sustained release of rhBMP-2 (Figure 1A,B). 

Notably, when observing the rhBMP-2 released from HA + TH over 1–3 weeks by concentration, when 2 μg was mixed, 43.49% was released in 0–1 weeks and 6.72% was released in 1–3 weeks. However, when 10 μg of rhBMP-2 was used, 37.13% was released in 0–1 weeks and 7.99% was released in 1–3 weeks (Figure 1C). Thus, about 50% of mixed rhBMP-2 was released in 1 week, and the release amount was modulated according to the amount of mixed rhBMP-2. This finding indicates the importance of mixing the right concentration of rhBMP-2 for more efficient fusion.

### 2.2. Appropriate Use of BMP-2 for Efficient Osteogenesis

**Figure 2:**
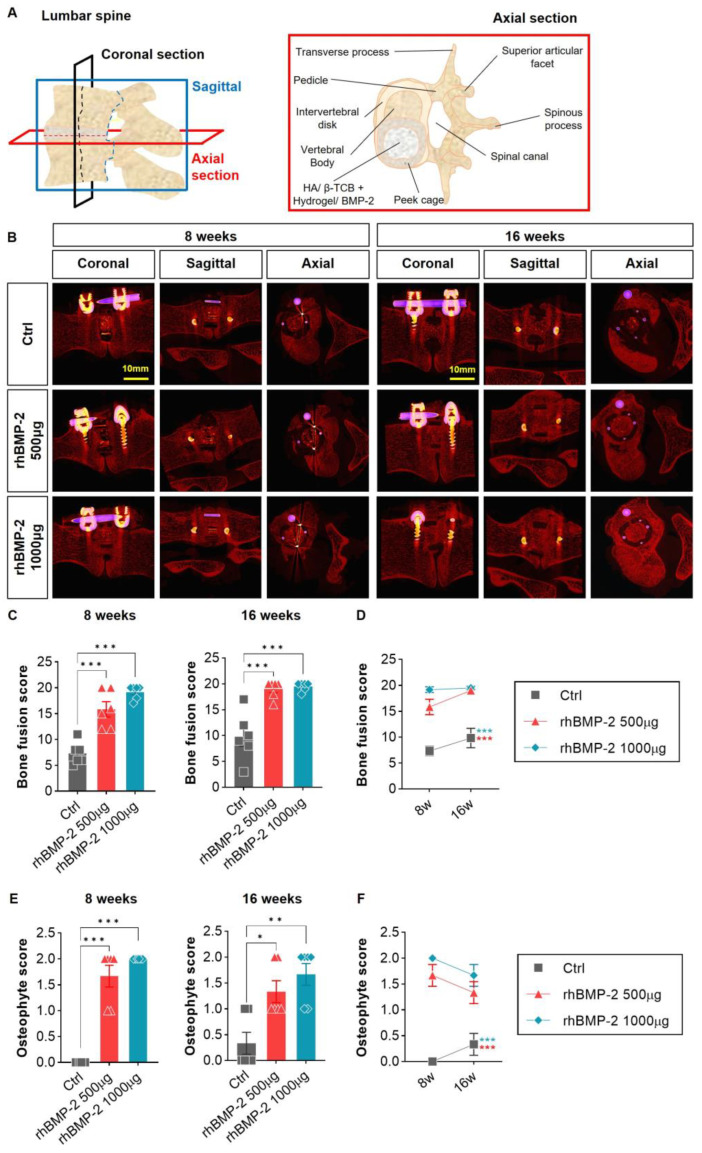
Fusion rate and osteophyte analysis of 2-dimensional sections. (**A**) A schematic image of the lumbar spine and cage implanted axial section. (**B**) Through micro-CT, the fusion rate was analyzed and scored from 0 to 20 points. (**C**,**D**) A significant difference in fusion was clearly observed between the control (Ctrl) and BMP groups. The BMP group had a higher fusion score. (**E**,**F**) A significant difference was also found in osteophyte formation, with lower BMP concentrations corresponding to lower scores. * *p* < 0.05, ** *p* < 0.01, and *** *p* < 0.001 indicate statistically significant differences.

At 8 weeks, the fusion rate score of the 500 μg rhBMP-2 group and the 1000 μg rhBMP-2 group was 15.83 vs. 19.17, indicating that the 1000 μg rhBMP-2 group had a higher fusion rate. Furthermore, at 16 weeks, the fusion rate between the 500 μg rhBMP-2 group and the 1000 μg rhBMP-2 group was 19.00 vs. 19.50. The fusion rate score was significantly higher in both groups than in the group without rhBMP-2, regardless of transplantation weeks. However, there was no statistical difference between the two rhBMP-2 groups (Figure 2C,D). Similarly, in the case of osteophytes, the osteophyte score of the rhBMP-2 group was statistically higher than the non rhBMP-2 group, but there was no difference between the rhBMP-2 groups (500 μg vs. 1000 μg, Figure 2E,F).

We also checked the fusion area and the osteophyte area in the Goldner’s trichrome (GT)-stained tissue samples from 16 weeks. When checking the cross-sectional length of both the bone fused inside the cage and the whole bone that grew to the outside of the cage, the shortest length was observed in the control group, while increases in length were observed in the groups containing 500 μg and 1000 μg of rhBMP-2. In particular, in the group where 1000 μg was mixed, the bone had proliferated to the outside of the cage, greatly exceeding the size of the block, which was made for the histology examinations. This excessive proliferation may irritate the spinal cord in the spinal canal, causing neuropathic pain and incomplete or complete paralysis [32].

### 2.3. Appropriate Using of BMP-2 for Activation of Trabecular Bone Formation

**Figure 3:**
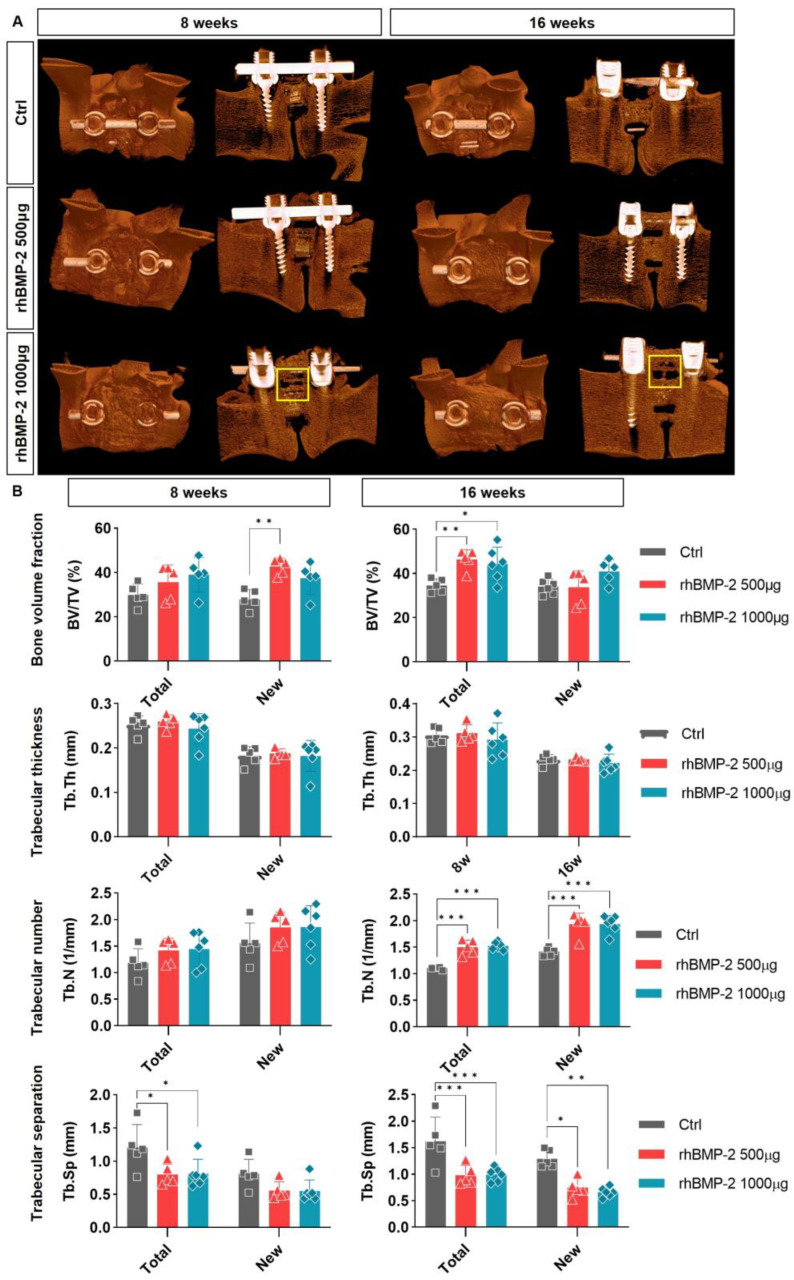
Trabecular bone analysis of 3-dimensional micro-CT images. (**A**) A 3D-reconstruction image of spine OLIF using micro-CT. (**B**) Trabecular bone analysis on a 3D-micro-CT image. The bone volume fraction, trabecular thickness, number, and separation were analyzed for total and new bone. In total and new bone, the bone volume fraction and trabecular number increased in proportion to the presence of rhBMP-2. However, fiber strain segregation showed an inverse pattern, decreasing with higher rhBMP-2 concentrations. However, significant differences were not found according to the concentration of rhBMP-2, and the low-rhBMP2-concentration group tended to show higher values for the bone volume fraction. * *p* < 0.05, ** *p* < 0.01, and *** *p* < 0.001 indicate statistically significant differences.

The 3D reconstructed pictures were first used to identify the sagittal surface of the screw-and-rod-fixed spine and the coronal plane for better visualization of the fusion sites. As shown in Figure 3, on the sagittal surface, we can observe not only the cage-implanted vertebral bodies, but also bone spurs (i.e., osteophytes), that had grown outward to cover the screw and rod. In this study, no osteophytes were observed in the groups containing 0 and 500 μg (1.9 mg/cc) of rhBMP-2, whereas bone spurs (yellow box) grew into the spinal canal site at both 8 and 16 weeks in the 1000 μg (3.8 mg/cc) group. Thus, osteophytes increased in proportion to the concentration of rhBMP-2 (Figure 3A).

Another analysis was performed in 3D reconstruction, but in order to distinguish the regenerated bone centering on the rhBMP-2 ± synthetic bone graft implanted inside the cage while connecting the upper and lower vertebral bodies, we analyzed the variables for the trabecular bone rather than the cortical bone. Four parameters were analyzed: the bone volume fraction (i.e., the volume of the total bone relative to the trabecular bone), trabecular thickness (i.e., the average thickness of the trabecular bone), trabecular number (i.e., the number of trabecular bones per unit length), and trabecular separation (i.e., the average distance between trabecular bones). In order to confirm the effects of the implanted artificial bone and rhBMP-2 on both the newly generated bone and the entire bone tissue of the animal, they were analyzed separately. The bone volume fraction tended to increase in proportion to the concentration of rhBMP-2 at both 8 and 16 weeks, with new bone at 8 weeks and total bone at 16 weeks showing statistical significance. However, a statistically significant difference was not observed between the rhBMP-2 500 μg and 1000 μg groups. Trabecular thickness was similar in all groups, with or without rhBMP-2, but the trabecular number showed a tendency to increase in proportion to the concentration of rhBMP-2. However, as with bone volume fraction, this value did not show statistical significance between the rhBMP-2 500 μg and 1000 μg groups, and there was little difference at 16 weeks. Conversely, the trabecular separation values showed an inverse pattern, decreasing as the rhBMP-2 concentration increased, and statistical significance was observed in both total and new bone at 8 and 16 weeks (Figure 3B). These results confirm that rhBMP-2 increases the overall bony volume, which tends to decrease the inter-bone spacing by increasing the number of bones rather than their thickness. This also means that not only the new bone, but also the existing bone around the implant site, will be affected; the total bone will therefore also show a difference. However, the lack of statistical significance in the difference between the rhBMP-2 500 μg and 1000 μg groups implies that increasing the concentration of rhBMP-2 beyond a certain threshold does not significantly affect fusion.

### 2.4. Appropriate Use of BMP-2 for Increased Bone Formation and Suppression of Inflammatory Response

**Figure 4:**
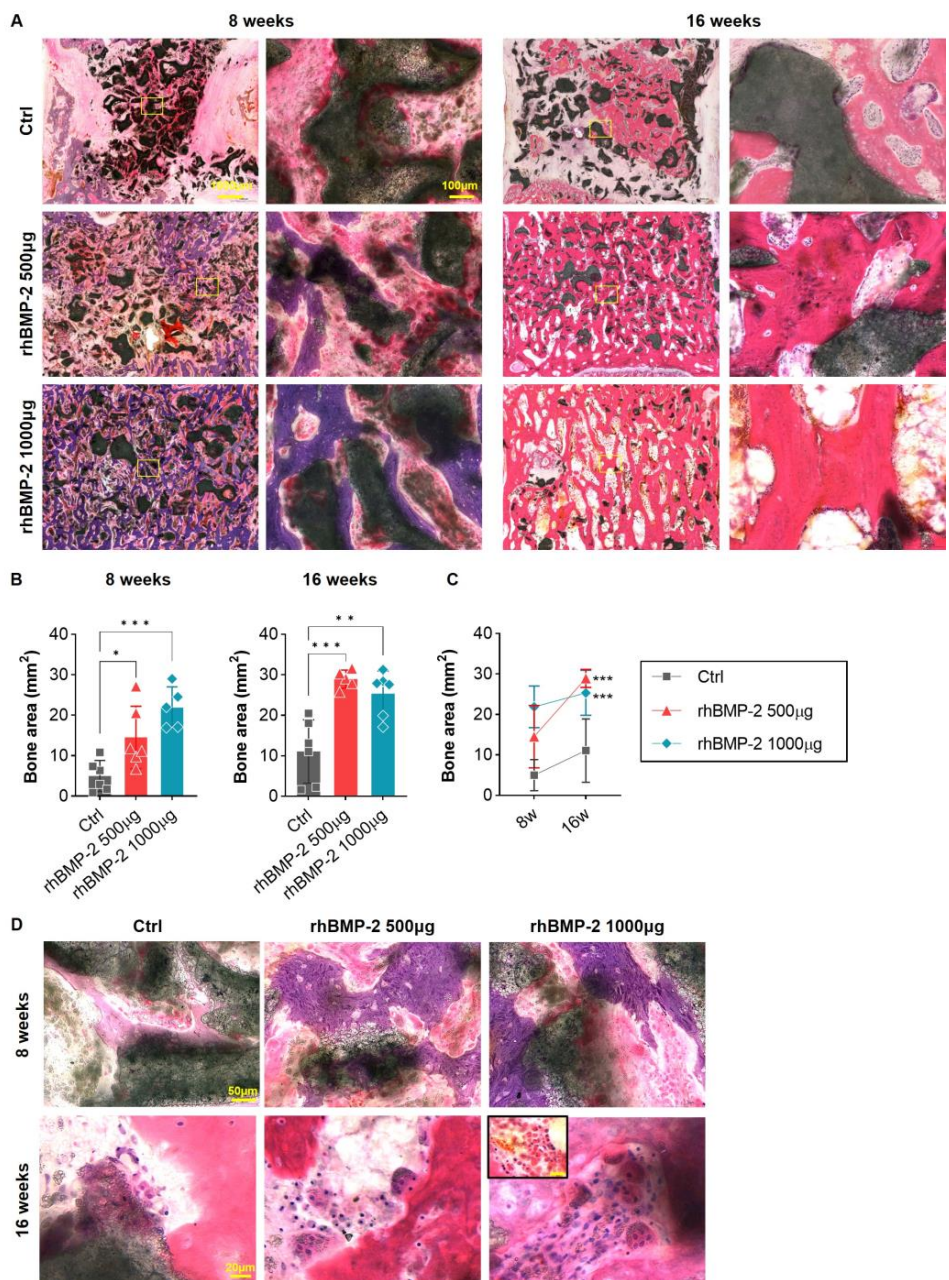
H&E staining to confirm the bone region and inflammatory reaction. (**A**) Overall and magnified pictures after hematoxylin and eosin (H&E) staining inside the cage of the fusion area. (**B**,**C**) Graphs analyzing the bone area (stained purple and pink). The bone area increased in proportion to the concentration of rhBMP-2 in the 8-week spine samples, but the low-concentration group (500 μg of rhBMP-2) showed higher values in the 16-week spine sample. (**D**) Magnified image of the fusion region. The BGM carrier was used for the synthesis of the new bone area at 8 weeks. Bone remodeling by osteoclasts was accelerated at 16 w after new bone synthesis was completed and increased in proportion to the concentration of rhBMP-2. Furthermore, inflammatory cells were recruited in the fusion region in the high-concentration group (1000 μg of rhBMP-2). * *p* < 0.05, ** *p* < 0.01, and *** *p* < 0.001 indicate statistically significant differences.

Fusion areas were observed after H&E staining at 8 and 16 weeks after OLIF. At 8 weeks in the control group, the putty was hardly used as bone material in the central part of the cage and remained there, and only a small part had changed to bone. Newly formed bone was observed mainly at the end plate of the vertebral body, where the cage was inserted. After 16 weeks, the putty had almost decomposed and the bone area had increased, but the level failed to show a significant change when compared with the group containing rhBMP-2. In the group where 500 μg of rhBMP-2 was mixed with putty, the bone area gradually increased, and rhBMP-2 exerted a continuing effect on the increased bone area for about 16 weeks. In the group where 1000 μg of rhBMP-2 was mixed with putty, the putty was depleted faster, large areas were replaced by bone, and consequently almost no putty remained at 16 weeks (Figure 4A,B). However, the increase in bone area from 8 to 16 weeks was not large compared to that observed in the group where the putty was mixed with 500 μg of rhBMP-2. One reason may be that bone remodeling occurs with osteoclastogenic rather than osteoblastic differentiation to increase the new bone area at 16 weeks. Furthermore, in the group mixed with 1000 μg of rhBMP-2, some individuals showed fibrosis at the fusion site, along with an inflammatory reaction, which may have reduced the overall rate of increase (Figure 4C).

### 2.5. Appropriate Use of BMP-2 for Organic and Inorganic Osteosynthesis

**Figure 5:**
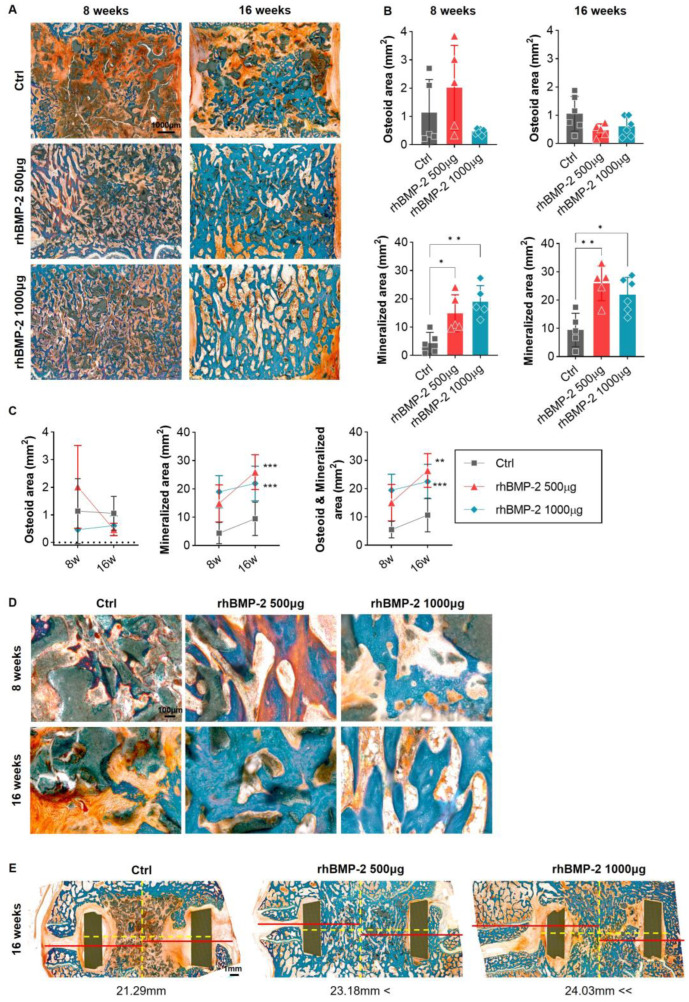
GT staining of organic and mineralized regions. (**A**) Overall and magnified pictures after GT staining inside the cage of the fusion area. (**B**,**C**) A graph analyzing the osteoid region (stained red and orange) and the mineralized region (stained sky blue). The low-concentration group (500 μg of rhBMP-2) had an overwhelmingly high value of the osteoid area at 8 weeks. It also showed high values of the mineralized region compared to the other group at 16 weeks. (**D**) Magnified images of the fusion region. In the control (Ctrl) group, the osteoid and mineralized areas formed in small proportions and were located between the BGM carrier. In the low-concentration group (500 μg of rhBMP-2), the osteoid and mineralized areas were mixed at 8 weeks, but the mineralized area increased overwhelmingly at 16 weeks. In the high-concentration group (1000 μg of rhBMP-2), only the mineralized area was observed at all periods. (**E**) Graphs analyzing the fusion rate and osteophytes. (**D**) Sagittal section of a lumbar spine from 16 weeks that was subjected to GT staining. Outside the PEEK cage, an increase in osteophytes was observed in proportion to the concentration of rhBMP-2. * *p* < 0.05, ** *p* < 0.01, and *** *p* < 0.001 indicate statistically significant differences.

After GT staining at 8 and 16 weeks after OLIF, fusion regions were observed separately in osteoid and mineralized bone areas. In the control group at 8 weeks, mainly putty and fibrous tissue were observed rather than the newly created bone area centering on the inside where the cage was inserted, and some osteoid and mineralized bone areas were observed between the areas of putty. After 16 weeks, there was a significant increase in the refined bone area, similar to the H&E stained bone area, but putty and fibrotic tissue were still observed in a high percentage. In contrast, in the rhBMP-2-mixed group, putty was depleted in the new bone areas and little fibrous tissue was observed. At 16 weeks, the remaining putty had almost disappeared, and the mineralized bone area had increased greatly. Notably, the rhBMP-2 500 μg group had twice the osteoid area at 8 weeks compared to the rhBMP-2 1000 μg group, and at 16 weeks the mineralized bone area had increased more than in the 1000 μg group (Figure 5). This means that rhBMP-2 induces more osteoblastogenesis when it is secreted in an appropriate amount than when it is secreted at a high concentration, whereas high concentrations induce less osteoblastogenesis and consequently less mineralization.

**Figure 6:**
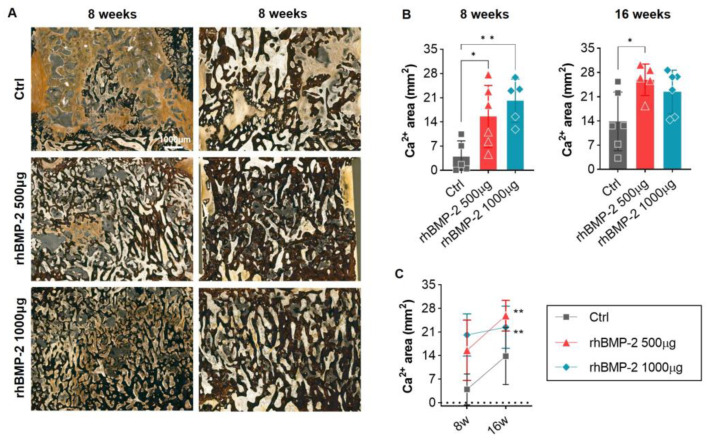
VK staining of calcium-positive mineralized regions. (**A**) Overall and magnified pictures after VK staining inside the cage of the fusion area. (**B**,**C**) A graph analyzing calcium-positive mineralized areas (stained dark brown). The calcium-positive area increased in proportion to the concentration of rhBMP-2 in the 8-week spine samples. However, the low-concentration group (500 μg of rhBMP-2) showed high values in the 16-week spine samples. * *p* < 0.05 and ** *p* < 0.01 indicate statistically significant differences.

We also identified calcium-positive bone areas after VK staining at 8 and 16 weeks after OLIF. In the control group, putty and fibrous tissue were mainly observed rather than calcium-positive bone area, which increased greatly at 16 weeks. However, this value was still lower than observed in the rhBMP-2 groups, with a difference of about 5 to 6 times or more at 8 weeks, and a difference of about 1.5 to 2 times at 16 weeks. As with the mineralized bone area in GT staining, the calcium-positive bone area was lower in the 500 μg group than in the 1000 μg group at 8 weeks, but at 16 weeks, the 500 μg group overtook the 1000 μg group (Figure 6).

## 3. Discussion

The manner in which rhBMP-2 is released in rhBMP-2-loaded artificial bones may have a significant effect on bone fusion. A previous study observing the release pattern of rhBMP-2-loaded carriers found that the collagen sponge, which was widely used in the past, released 77.05% of the rhBMP-2, while the TH released 20.18%, in 1 week. After 3 weeks, an additional 1.35% was released from the collagen sponge, while the TH released 2.35% of the rhBMP-2. Thus, 77.5% of the rhBMP-2 remained in the artificial bone, indicating sustained release [27]. However, rhBMP-2 is expected to be released more slowly in vivo than in vitro because rhBMP-2-loaded artificial bones are located in confined spaces in cages and vertebral bodies under in vivo conditions. However, some studies have shown that loading high concentrations of rhBMP-2, similar to the use of collagen sponges, may result in the early release of a large amount of rhBMP-2, leading to inflammation in structurally abnormal bones and implantation sites [33]. Therefore, using rhBMP-2 above a certain threshold concentration may not induce bone fusion and healing, but rather promote lower-quality bone. In this study, we investigated the rate of increase in organic and inorganic bone tissue between groups loaded with different rhBMP-2 concentrations. The group without rhBMP-2 showed a much lower fusion rate than the group with rhBMP-2, but the group loaded with 1000 μg had higher values at 8 weeks than were observed in the 500 μg group. Nevertheless, at 16 weeks, the group loaded with 500 μg showed higher values, showing a reversed pattern over time.

As osteogenesis accelerates, the proliferation and differentiation of osteoblasts increase, and rhBMP-2 is the most important molecule in mesenchymal stem cells, proceeding through osteoprogenitor cells to the final mature osteoblasts [34,35]. Matrix vesicles have several membrane transporters and enzymes associated with mineralization, thus providing a microenvironment for the formation of calcium phosphate crystals and the growth of HA [Ca_10_(PO_4_)_6_(OH)_2_] crystals. This occurs as Ca^2+^ binds strongly to the inner leaflet of the plasma membrane, which is generally negatively charged, and binds strongly to phosphatidylcholine and phosphatidylserine in the leaflet [36,37]. HA crystals are then formed, which penetrate the plasma membrane to give rise to mineralized nodules [38]. The mineralization of the collagen deposition of calcium phosphate begins from the mineralized nodules that are produced, forming and completing grains [39]. Osteocytes differentiated from osteoblasts extend dendrites and transmit bone remodeling signals, resulting in the alignment of collagen fibers and communication with osteoblasts and bone lining cells [40,41]. Thus, we found that the BMP-2-loaded putty, containing HA and TCP, provided a basis for initiating these mineralization processes, and the secreted rhBMP-2 led to the differentiation and maturation of osteoblasts, facilitating their transition to osteocytes.

In various studies, the hydrogel has shown various therapeutic effects such as angiogenesis, osteogenesis, with inflammatory reaction avoided [42,43]. It provides an appropriate microenvironment for recovery-related cells such as endothelial cells, osteoblasts, and mesenchymal stem cells to migrate because of its synthesis method and degradation rate regulation [44]. We used a hydrogel polymer to sustain the release of BMP-2 and regulate the degradable rate of the ceramic carrier HA and b-TCP. The hydrogel polymer was formed from a b-TCP/hydrogel putty. This putty form can be conveniently mixed with other materials in the desired form [27]. In addition, these putties not only have osteogenesis properties but also a hemostasis effect with angiogenesis at the injury site [44]. These newly developed carriers sustain the release of the growth factor which is rhBMP-2 by slow degradation as shown in a former study [27,45]. 

In conclusion, the BMP-2-loaded TH carrier used in this study showed a sustained release of BMP-2, overcoming the drawback of potentially causing side effects when used at high concentrations. This system may also be an efficient option, since it provides mineral matter to accelerate the fusion rate of the spine and increase bone quality.

## 4. Materials and Methods

### 4.1. Preparation of the Composite Carrier

The mixed bone graft material (BGM; CGBio Co., Ltd., Seongnam-si, Gyeonggi-do, Republic of Korea) consisted of porous HA granules, TH, and rhBMP-2. rhBMP-2 was dissolved in water for injection at 2.5 mg/mL and absorbed into the porous HA granules. The BGM was completed by mixing the TH into a syringe containing rhBMP-2-absorbed HA granules. The hydrogel, which is one component of TH, is based on poloxamer 407 and hydroxypropyl methylcellulose (HPMC). Poloxamer 407 has thermo-sensitivity, usually used in dental surgery [46]. HPMC is usually used as a drug tablet or soft gel capsule as a controlled release matrix [47,48]. All materials were prepared by mixing immediately prior to use while maintaining sterility. The ratio of HA to TH was 1:2 (wt%), and the final formulation was putty for injection. The final concentrations of rhBMP-2 per graft volume were 0, about 1.9 mg/cc and 3.8 mg/cc. 

### 4.2. ELISA Assay

An enzyme-linked immunosorbent assay (ELISA) was performed to confirm the rhBMP-2 release pattern according to the composition of the BGM. Specifically, 0, 2, and 10 μg rhBMP-2 were mixed with HA, TH, and HA + TH, respectively, and placed in a 6-well insert system, followed by filling the well plate with phosphate-buffered saline (Hyclone, Logan, UT, USA). The secretion of rhBMP-2 across the membrane was quantified at 1, 3, 6, 9, 12, 18, 24 h and 1, 2, 3 weeks. Quantitation was performed according to the defined specifications of the human BMP-2 ELISA kit (RHF913CKX, Antigenix America Inc., Melville, NY, USA). The measurements were read at 450 nm on a Microplate Reader (Synergy HTX Multi-Mode Microplate Reader, BioTek Instruments, Winooski, VT, USA).

### 4.3. Mini-Pig Oblique Lateral Interbody Fusion Model

**Figure 7:**
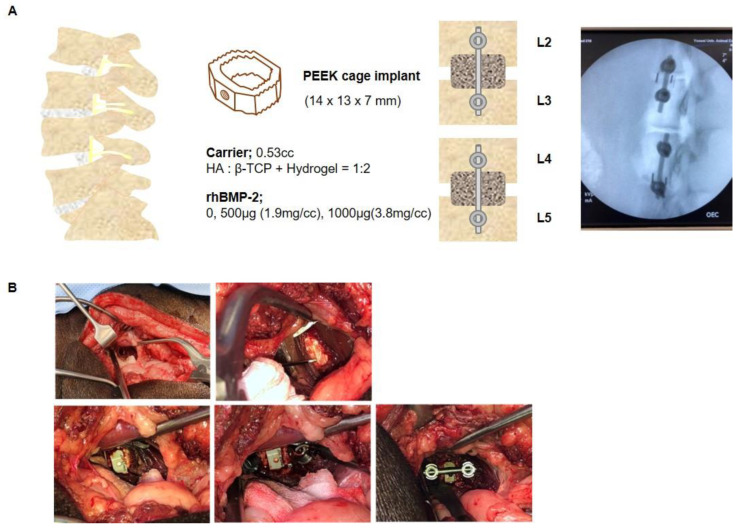
Establishment of the swine OLIF model. (**A**) Schematic and X-ray images of the swine oblique lateral interbody fusion (OLIF) in the lumbar spine. rhBMP-2 (0, 500 μg [1.9 mg/cc]), 1000 μg [3.8 mg/cc]) was mixed with 0.53 mL of the bone graft material (BGM). The BGM was used to fill the PEEK cage (14 × 13 × 7 mm) and implanted at the lumbar 2–3 and 4–5 levels. (**B**) Steps of OLIF surgery in the swine lumbar spine.

In this study, a total of 18 female mini-pigs (Sus scrofa domestica, mean ± standard deviation weight: 50 ± 5 kg; CRONEX M-Pig, CRONEX Co., Ltd., Seongnam-si, Gyeonggi-do, Republic of Korea) were divided into a total of 3 groups, and oblique lateral interbody fusion (OLIF) was performed at 4 levels and 2 sites per individual. All animals were housed 4–5 per cage and supplied with an appropriate diet (Cromeal, CRONEX Co., Ltd.) of 750 g per 12 h. The drinking water was filtered, and UV-sterilized underground water was freely ingested through an automatic water supply system. The animals were maintained at a temperature of 20–24 °C, relative humidity of 50 ± 20%, ventilation holes of 10–15 per hour, light/dark intervals of 12 h, and illuminance of 150–300 Lux until they were transferred for surgery. Animal surgery and housing were performed in accordance with the Association for Assessment and Accreditation of Laboratory Animal Care (AAALAC)-stipulated regulations, and all experiments were managed and supervised with permission from Institutional Animal Care and Use Committee (IACUC; protocol number: 2019-0061). The mini-pigs were placed under anesthesia with ketamine (20 mg/kg; Yuhan, Seoul, Republic of Korea), Rompun (2 mg/kg; Bayer, Leverkusen, Germany), and after that maintained with Isoflurane USP 2% (Isotroy 100, Troikaa Pharmaceuticals Ltd., Gujarat, India). The animals were fixed in the lateral position before surgery, and the target level was confirmed by C-ram imaging. The muscles were separated from the vertebral body of the targeted spine levels, and enough space was secured to tighten the screws, including the intervertebral disc (or intervertebral fibrocartilage). Touching or cutting the spinal nerve that comes out of the spinal cord can cause paraplegia, so a high degree of caution was required. The discs were removed between the target lumbar levels (2–3, 4–5) and drilling was performed to shave off some of the exposed vertebral body end plates. Polyetheretherketone (PEEK) cages (CGBio Co., Ltd.) were implanted with BGM in the interbody space (Figure 7A,B). In order to increase the fusion rate, a titanium screw (GS Medical, Cheongwon-gun, Chungcheongbuk-do, Republic of Korea) was driven into the vertebral body and fixed to a rod (GS Medical). The muscles were sutured, and an antibiotic (cefazoline, 15 mg/kg; Chong Kun Dang Pharmaceutical Corporation, Seoul, Republic of Korea) and a painkiller (Meloxicam 0.2 mg/kg; Ourofino, São Paulo, SP, Brazil) were administered for 1 week. The lumbar vertebral body, including the fusion region, was then obtained at 8 weeks and 16 weeks for imaging and histological analysis.

### 4.4. Fusion and Osteophyte Analysis

#### 4.4.1. Fusion Rate

In total, 10 images were analyzed at 1 mm intervals across the cage inner space with the sagittal aspect perpendicular to the bone endplate. The fusion rate between the new bone and the endplate in the inner cage space was checked, and if the trabecular bone was well formed and the bridge accounted for more than 50% of the length, a score of 1 was given, while if it was less than 50%, a score of 0 was given. Therefore, a minimum score of 0 to a maximum of 20 points was given per specimen.

#### 4.4.2. Osteophyte

Similarly, sagittal computed tomography (CT) images of the disc spaces into which the implants were placed were analyzed. A representative image showing the largest osteophyte was selected. Most osteophytes were visually identified in a gross view. However, raw osteophyte specimens could not be obtained without sacrificing the mini-pigs, so it was not possible to conduct accurate numerical comparisons. Therefore, we decided to use a 2-point system based on whether the osteophyte seen in the disc space was larger than the diameter of the cage. If the osteophyte was less than 50% of the diameter of the cage, a score of 1 was given. If the osteophyte was more than 50% of the cage, its score was 2 points, then, if an osteophyte was not clear on the CT image, it was scored as 0. The osteophyte score was measured from 0 to 2 points.

### 4.5. Micro-CT Analysis

All animals were sacrificed by cutting the aorta at 8 or 16 weeks and exsanguination; then, lumbar vertebrae 2–3 and 4–5 were separated and fixed in 10% formalin solution (Merck, Darmstadt, Germany) at room temperature for 7 days. The spine samples were scanned with Skyscan1173 micro-CT imager (Bruker-CT, Kontich, Belgium) and image control software (version 1.6, SkyScan 1173, Bruker-CT). Scanning was performed at an energy of 130 kVp and current of 60 µA with a 7.1-µm voxel resolution using 2240 slices. The raw image data were 3D reconstructed using CTVOX (Ver. 3.3.0, Bruker-CT). The reconstructed images were analyzed using CTAn Software (ver. 1.19.40., Bruker-CT). The analysis focused on two phenomena of interest: (1) fusion and osteophytes, and (2) cancellous bone at the fusion site.

### 4.6. Sample Preparation

A pretreatment step was necessary for special staining of non-decalcified tissue. First, the tissue was immersed in 70% ethanol (Merck) for 2 days, 95% ethanol for 2 days, and 100% ethanol for 3 days to dehydrate. The specimens were then embedded for 3 days in Technovit 7200 (VLC, Heraeus Kulzer, Wehrheim, Germany), a component of a resin based on light-curing methacrylate. The sample was polymerized using a photopolymerization apparatus (Exact 520, Exact, Norderstedt, Germany), and care was taken that the temperature did not exceed 40 °C. The embedded sample was cut to a certain size around the fusion site and attached to an acrylic slide. It was then cut to a thickness of approximately 300 μm using a cutting band (Exakt 300, Exact) and re-ground to 60 μm with the same machine and sandpaper before staining.

### 4.7. H&E Staining

Samples were stained with Mayer’s hematoxylin (Merck, Darmstadt, Germany). for 10 min and washed with tap water for 10 min. Next, samples were stained with 1% alcohol Eosin Y (Merck) for 10 s, and then dehydrated with 70 and 95% ethanol once each and 100% ethanol twice. Slides were then mounted using a permanent mounting medium (Permount Mounting Medium, Electron Microscopy Sciences, Hatfield, PA, USA).

### 4.8. Goldner’s Trichrome Staining

Samples were stained with Weigert’s iron hematoxylin (Merck) for 5 min and washed with tap water for 10 min. Then, after bluing with a ponceau acid fuchsine solution (Sigma-Aldrich, St Louis, MO, USA) for 5 min, they were washed with 1% acetic acid (Sigma-Aldrich) for 1 min. Samples were decolorized with Orange G solution (Merck) for approximately 5 min until the collagen was destained and washed again with 1% acetate for 1 min. Finally, after being treated with a light green solution (Histo-Line Laboratories, Pantigliate, Milano, Italy) for 10 min, samples were thoroughly washed with tap water. They were dehydrated using 70 and 95% ethanol once each and 100% ethanol twice, and mounted using a permanent mounting medium.

### 4.9. Von Kossa Staining

Before staining, samples were hydrated with distilled water, immersed in a 10% silver solution (Sigma-Aldrich) and placed in an oven at 70 °C for 20 min. After that, 1–3 drops of 10% silver solution were applied and left in EXAKT 520 (Gen-R-103, Exact) for about 30 min until the calcium on the sample turned black. After washing with tap water and rinsing with distilled water, 1 to 3 drops of 5% sodium thiosulfate (Sigma-Aldrich) were added dropwise and allowed to react for 3 min. After washing again with tap water and rinsing with distilled water, 1 to 3 drops of nuclear fast red solution (Sigma-Aldrich) were dropped to react on the slides, and then washed with tap water. Finally, after dehydration with 70, 95 and 100% ethanol, the slides were mounted using mounting medium.

### 4.10. Slide Scanning and Image Analysis

All stained slides were scanned using a digital slide scanning automation system (Pannoramic 250 FLASH III, 3D HISTECH, Sysmex, Budapest, Hungary), and the images were processed using CaseViewer (3D HISTECH, Sysmex).

### 4.11. Statistical Analysis

All data are presented as means± standard deviation. One-way analysis of variance with multiple comparisons and Tukey’s test (*, *p* < 0.05; **, *p* < 0.01; and ***, *p* < 0.001) were performed. A *p*-value of <0.05 was considered statistically significant. GraphPad PRISM 9.4.1 (GraphPad Software Inc, San Diego, CA) was used to generate graphs and conduct the statistical analysis.

## Data Availability

The data presented in this study are available in this article.
